# Rise and fall of sea ice production in the Arctic Ocean’s ice factories

**DOI:** 10.1038/s41467-022-34785-6

**Published:** 2022-12-17

**Authors:** S. B. Cornish, H. L. Johnson, R. D. C. Mallett, J. Dörr, Y. Kostov, A. E. Richards

**Affiliations:** 1 Department of Earth Sciences, University of Oxford, Oxford, UK; 2 Centre for Polar Observation and Modelling, Earth Sciences, UCL, London, UK; 3 Geophysical Institute, University of Bergen, Bergen, Norway; 4 Bjerknes Centre for Climate Research, Bergen, Norway; 5 College of Life and Environmental Science, University of Exeter, Exeter, UK

**Keywords:** Physical oceanography, Cryospheric science, Climate and Earth system modelling

## Abstract

The volume, extent and age of Arctic sea ice is in decline, yet winter sea ice production appears to have been increasing, despite Arctic warming being most intense during winter. Previous work suggests that further warming will at some point lead to a decline in ice production, however a consistent explanation of both rise and fall is hitherto missing. Here, we investigate these driving factors through a simple linear model for ice production. We focus on the Kara and Laptev seas-sometimes referred to as Arctic “ice factories” for their outsized role in ice production, and train the model on internal variability across the Community Earth System Model’s Large Ensemble (CESM-LE). The linear model is highly skilful at explaining internal variability and can also explain the forced rise-then-fall of ice production, providing insight into the competing drivers of change. We apply our linear model to the same climate variables from observation-based data; the resulting estimate of ice production over recent decades suggests that, just as in CESM-LE, we are currently passing the peak of ice production in the Kara and Laptev seas.

## Abstract (web summary)

Winter sea ice production appears to have been increasing, despite Arctic warming being most intense during winter. Here the authors examine the competing factors controlling sea ice production in the Kara and Laptev seas, and develop a simple model that explains the rise and subsequent fall of ice production under climate change.

## Introduction

Arctic sea ice extent and thickness is in an on-going decline, sustained over at least the length of the satellite record, and directly related to anthropogenic carbon emissions^1^. The retreat of Arctic sea ice is both an expression and driver of *Arctic Amplification*, which is the phenomenon of intensified climate change in the Arctic region relative to lower latitudes^2–4^. Though losses are observed in all seasons, they are more pronounced during late summer than late winter, in terms of both extent^5^ and thickness^6^. These trends reveal that increases in summer melting are to some extent compensated by increasing ice production during winter, though not by enough to prevent the continued decline of the annual mean reservoir of sea ice^6^.

Ice production is important for a number of reasons. As progressively more ice melts during the summer, winter ice production is critical in restoring the ice pack before the onset of polar day, when the high albedo of sea ice and its snow cover plays a crucial role in the radiative budget of the region. The distribution of sea ice is important during the winter; it limits heat fluxes from the ocean to the atmosphere and plays a complex part in the surface momentum balance^7^. Sea-ice growth also plays a vital hydrographic role: brine is rejected from sea ice during freezing, and freshwater is redistributed via sea ice motion between locations of growth and melt^8–10^.

There is an apparent tension in the observation of rising winter sea ice growth in concert with Arctic Amplification, which is intensified during the winter^3,11^. However, the two phenomena are most likely linked^12^. As progressively less sea ice survives the summer, the winter ice pack is increasingly thin, promoting both higher winter growth rates^13^ and increased heat fluxes from ocean to atmosphere^14^. Heat sequestered in the Arctic Ocean during summer is released into the atmosphere prior to and during winter sea ice growth^11^. Meanwhile, the sea ice growth rate is tightly coupled to heat fluxes from the ocean to the atmosphere, because it is determined by the energy balance at the lower boundary of the ice. As ice thickens, the conductive heat loss to the atmosphere declines, and growth rate slows in direct proportion. Sea ice thinner than ~0.4 m permits heat fluxes one to two orders of magnitude larger than those through perennial, thicker ice^15^. Lang et al.^14^ attribute a rise of ~1 °C in Arctic surface air temperature per decade to declining winter sea ice thickness. In principle, this surface warming may be partially offset by increased long-wave emission^16^. However, due to the predominantly stable atmospheric stratification, surface-intensified warming is inefficiently transmitted to the top of the atmosphere, and therefore outgoing long-wave emissions only very weakly compensate the warming (the *lapse-rate feedback*)^17–19^.

The inverse relationship between sea ice thickness and growth rate underpins a well-known negative feedback on Arctic sea ice loss^20^. There are other effects, however, that also act to promote winter sea ice growth in a warming Arctic. Sea ice that is formed later in the season is more likely to avoid the limiting effects of snow—an effective insulator—on growth rate^21,22^. Similarly, snow melt during summer preconditions more rapid growth at the beginning of the freezing season due to the loss of this insulating snow cover^23^. Additionally, thinner sea ice is weaker and more mobile, leading to an increase in wind-driven sea ice divergence^24–26^, which promotes winter growth^27^. These negative feedbacks provide stability to the Arctic sea ice system, such that rapid or irreversible losses in summer sea ice area are unlikely^16,22,28^.

In recent warm winters such as 2015/2016 and 2016/2017, growth suppression and even winter melting have been observed^13,29–31^, calling into question whether the action of these negative feedbacks may be becoming overwhelmed by warming. Based on simulations with the sea ice model CICE, Stroeve et al.^13^ suggest that the thermodynamic growth during the winter 2016/2017 was 11–13 cm lower than the 2011–2017 mean, with the overall positive trend in winter ice growth from 1985 ending in 2012. Winter ice growth then weakened until the end of the model run in 2018. Using the same large ensemble of coupled climate model runs we employ here (CESM-LE), Petty et al.^32^ identify that the positive correlation between temperature and winter growth weakens through the mid-century under the RCP8.5 emissions scenario, eventually becoming a negative relationship—indicative of warming overwhelming the negative feedbacks on sea ice loss. Observational evidence of increasing winter sea ice production, and model-based suggestions of an imminent or recently-begun decline—both associated with anthropogenic climate change—motivate a physical explanation of this rise-then-fall behaviour.

**Fig. 1:**
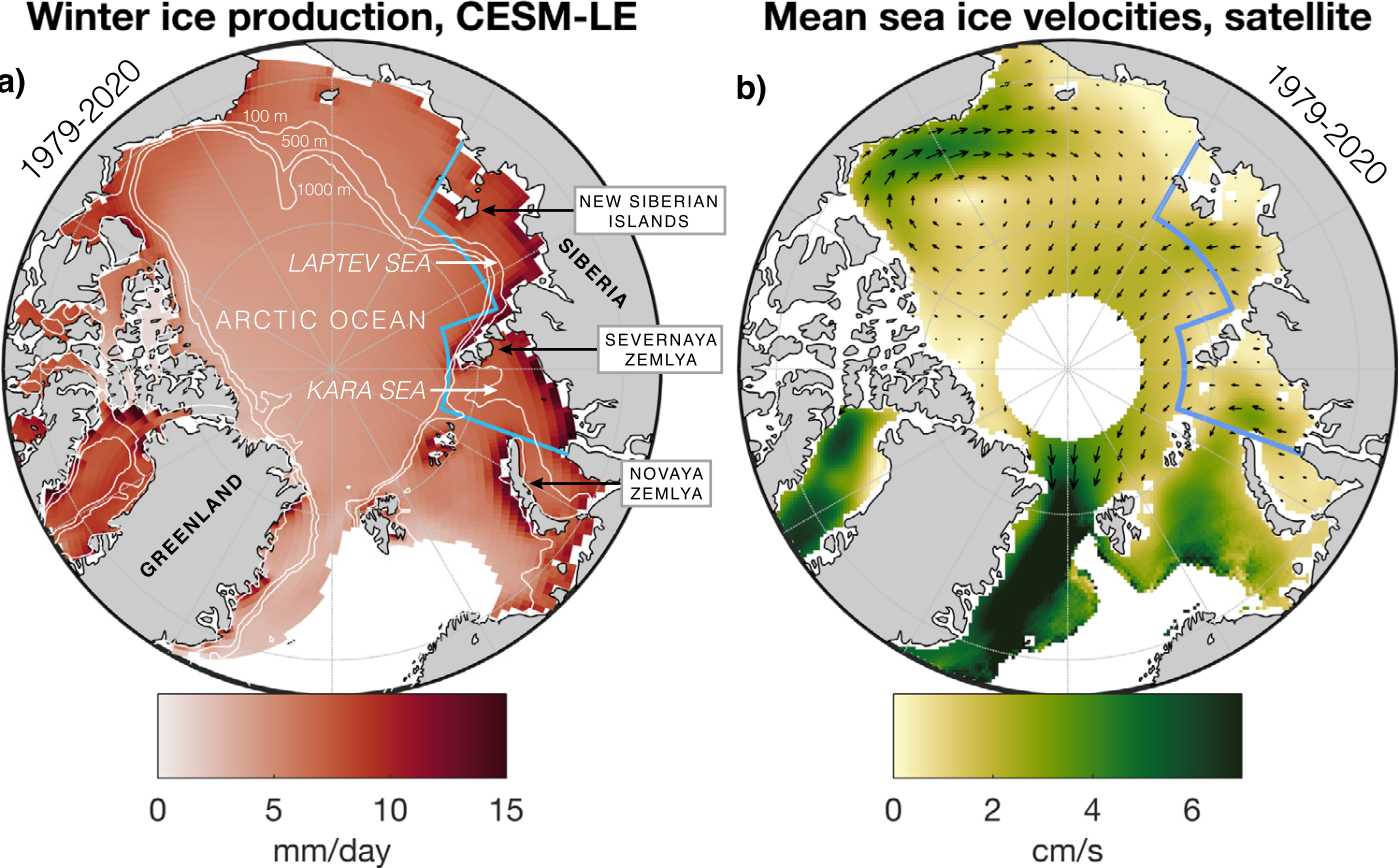
The Kara and Laptev seas: ice factories of the Arctic Ocean. **a** Winter (Oct-Apr) mean ice production for the period 1979--2020 in the ensemble mean of CESM-LE. The Kara and Laptev seas study region is outlined in blue. Bathymetric contours (100 m, 500 m, 1000 m) from CESM-LE shown in white. **b** Mean sea ice velocities during 1979--2020 as recorded by the Polar Pathfinder v4.1 product, gridded at 25 × 25 km resolution and in weekly means^88^. Only periods when sea ice is present are considered in the mean value for each grid cell. Shading indicates the magnitude of the mean velocities. Arrows are omitted in the Barents Sea, Baffin Bay and Greenland Sea, where velocities are significantly higher (colourscale saturates) and flow is generally southwards.

The Kara and Laptev shelf seas have been referred to as the “ice factories” of the Arctic Ocean due to their outsized contribution to Arctic sea ice production^33^ (Fig. 1a). Classified as interior shelves of the Arctic^34^, the Kara and Laptev seas are relatively fresh, receiving 50% of the freshwater runoff to the Arctic Ocean^35^. During winter, winds predominantly drive sea ice northwards from the Siberian coastline in the Kara and Laptev seas (Fig. 1b), opening up perennial flaw leads/polynyas, where open water separates landfast ice from mobile pack ice. Sea ice forms rapidly in these polynyas, and a number of studies have sought to quantify the contribution to the Arctic sea ice budget made by these regions^36–41^. The occurrence of wind patterns that increase the advection of sea ice away from the Siberian coasts thus intensifies sea ice production in these regions, and increases the fraction of (relatively thin) sea ice in the Arctic basin originating in the Kara and Laptev seas^42,43^. High rates of freezing in these shallow seas are also thought to help bolster the cold halocline (salinity-dominated stratification) of the Arctic Ocean^44,45^. Sea ice produced in the Kara and Laptev seas freshens the ocean surface of the Eurasian Basin and northern Barents Sea upon melting during summer, helping to maintain the stratification that limits upwards heat fluxes from Atlantic Waters. The stability of the Arctic halocline has emerged as a key climate change indicator^46^, in light of episodic collapses of the winter halocline in the Eurasian basin and northern Barents Sea^47,48^, which lead to the shoaling of Atlantic Waters and increased heat fluxes to the surface^49^.

In this study, we develop a simple linear model for ice production that is informed by the physics of sea ice growth. The simple model can successfully explain both the rise and fall of ice production in the Kara and Laptev seas, as represented in the historical simulations and projections of a state-of-the-art global climate model. Our linear model thus offers insight into the competing processes at play under climate change. We further apply our linear model to observation-based data to estimate historical ice production. This estimate of historical ice production suggests that, as in the global climate model, the Kara and Laptev seas may presently be passing peak ice production.

## Results

### Shrinking growth season, rising growth rate

In our analysis of ice production in the Kara and Laptev seas we use 40 ensemble members from CESM-LE. Each ensemble member consists of a 20th Century run (20C) from 1920 to 2005 that employs historical external forcing and a ‘high-emissions’ RCP8.5 run from 2006 to 2080. Ensemble members are initialised in 1920 with small differences in atmospheric properties, which cause the simulations to evolve differently through time. We use this internal variability (the deviations of each ensemble member from the ensemble mean) across all 40 ensemble members to develop our linear model.

**Fig. 2:**
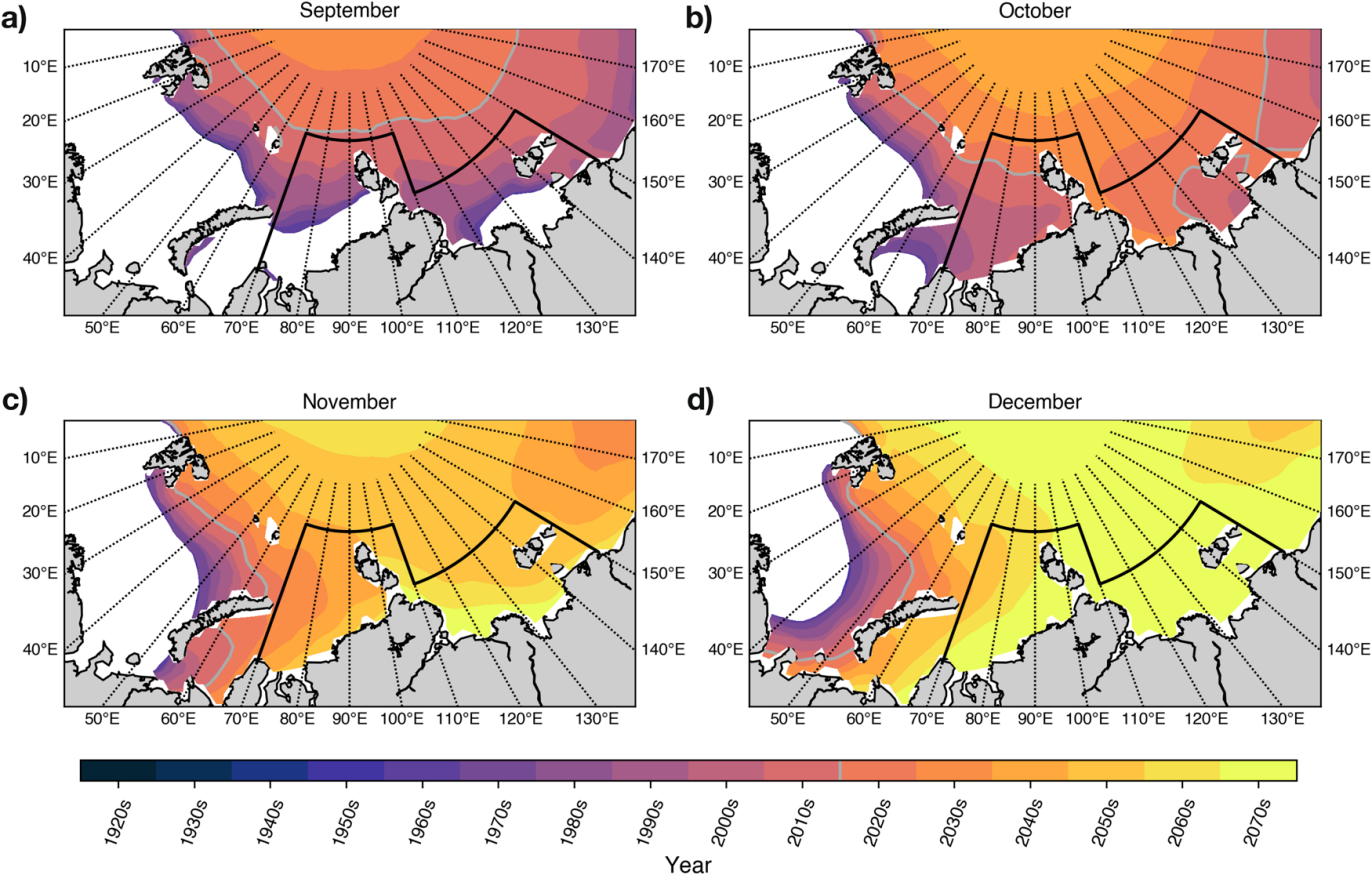
Changing sea ice extent during freeze-up. Decadal mean winter sea ice extent from the CESM-LE ensemble mean from 1920 to 2080, in (**a**) September, (**b**) October, (**c**) November and (**d**) December. Study region outlined in black. Ice edge defined at 15% concentration. Grey line marks the mean 2020s sea ice extent.

During the summer melt season, large areas of open water develop in the Kara and Laptev seas as the sea ice edge retreats towards the pole (Fig. 2a). The winter refreeze, however, begins along the Siberian coastline^50^. In CESM-LE, the newly formed ice along the Siberian coastline reconnects with the sea ice of the interior Arctic Ocean in an hourglass shape that becomes progressively pinched, and occurs later, through the 21st Century (Fig. 2).

**Fig. 3:**
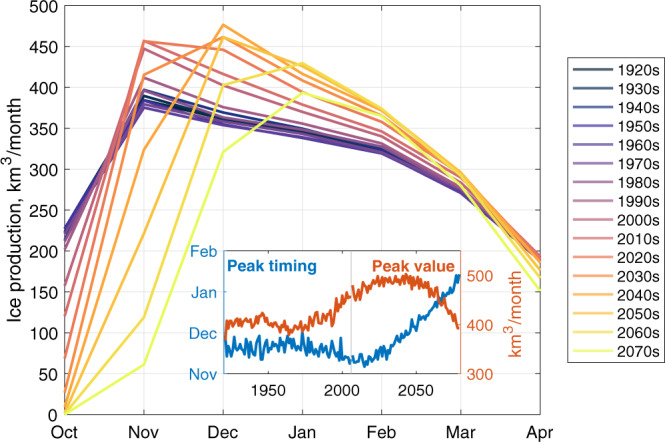
Changing winter cycles of ice production. Decadal ensemble mean sea ice production through winter months in CESM-LE. Inset panel shows changes in the timing of peak sea ice production and changes in the amplitude of the peak value. Note that the peak timing is interpolated using the mean of the 40 monthly resolution values from individual ensemble members.

On average, this refreezing in the early winter is the most productive period for winter ice growth (Fig. 3), and is linked to the areal expansion of sea ice (Fig. 2). After an initial peak, ice production in the ensemble mean gradually curtails through the winter—as ice and overlying snow cover thicken—before diminishing more steeply in April. As the climate warms in CESM-LE, however, October ice production rapidly falls, approaching zero by the 2030s (Fig. 3). Initially this trend is compensated by a rapid recovery and a higher sea ice production peak in November, connected to the refreezing of a larger open water area (c.f. Fig. 2). The peak in sea ice production shifts later in the year after the 2010s, continuing to increase in magnitude until the 2040s, before falling rapidly to mid 20th Century levels by 2080 (Fig. 3).

**Fig. 4:**
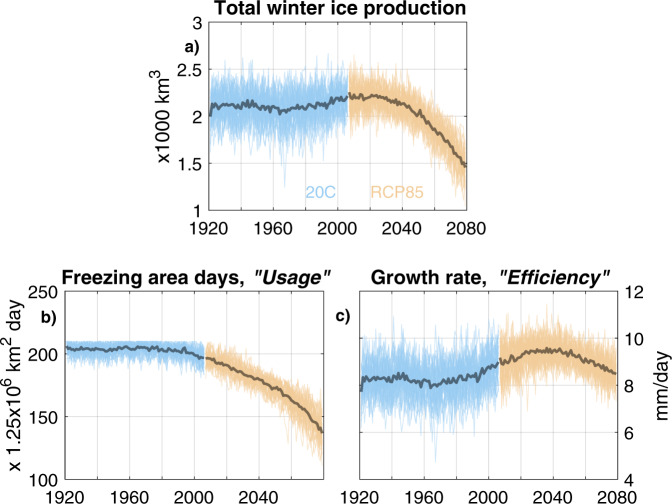
Decomposing winter ice production. Winter ice production (**a**) and decomposition into freezing area days (**b**) and growth rate (**c**). All 40 ensemble members shown in light blue (20C) and orange (RCP8.5). Ensemble-mean indicated by darker lines. Note that the freezing area days is rescaled in terms of the total area of the study region, such that the upper limit is 210 on the y-axis: the duration of our seven month winter period in days.

The overall trend is one of progressively delayed freezing onset, followed by ice production that is more intense in the mid-winter throughout RCP8.5 than it is in the 20th Century. The graph-area under the ice production curves in Fig. 3 represents the total winter ice production, our quantity of interest. In the ensemble mean, total ice production rises gently from approximately 1970 to 2010, before falling from 2020 onwards (Fig. 4a).

Examining the causes behind this rise and fall, we start by recognising that sea ice production is limited by the (location-dependent) duration of the freezing season, and the rate of growth where and when it is freezing. Indeed, we can decompose total ice production into these two components (Fig. 4). Firstly, the spatio-temporal duration of freezing, expressed as the number of freezing area days (Fig. 4b). To extend the ice factory metaphor, this can be viewed as the “usage” of the ice factory: the area and time over which it is producing sea ice (Eq. 1). Secondly, the mean growth rate over the regions where, and times when, sea ice is growing (Fig. 4c). In the ice factory metaphor, this is the “efficiency” of the ice factory where and when it is “in use”, and we calculate it simply as the total winter ice production divided by the number of freezing area days following Eq. 1:

1 $$\,{{\mbox{Ice factory production}}}\,\,({{{{{{{{\rm{m}}}}}}}}}^{3})\,=\,{{{{{{{\rm{usage}}}}}}}}\,({{{{{{{{\rm{m}}}}}}}}}^{2}{{{{{{{\rm{s}}}}}}}})\,\times\, {{{{{{{\rm{efficiency}}}}}}}}\,({{{{{{{{\rm{ms}}}}}}}}}^{-1})$$

The decomposition reveals quite different trends. The mean number of freezing area days (or ice factory usage), begins to decline from the 1990s onwards, and then accelerates after 2050 (Fig. 4b). The mean growth rate (or ice factory efficiency), on the other hand, is relatively elevated during the RCP8.5 run in comparison to the 20C run (Fig. 4c). Growth rate rises from approximately 1970 to 2030, before declining from approximately 2040 through to 2080.

Physically, the ice factory usage during Oct-Apr is controlled principally by the (location-dependent) time taken to cool the upper ocean to the freezing temperature (approximately −1.8 °C). Heat accumulates in the upper ocean during summer due to penetrating solar radiation, and is sequestered within and often below the mixed layer. In the early winter, ice growth is limited by upwards oceanic heat fluxes as mixing removes heat from the (deepening) mixed layer. A simple regression model, using the mean Sep surface ocean temperature as a single explanatory variable, captures the internal variability in the number of freezing area days with *R*^2^ = 0.74 during the 20C run, and with *R*^2^ = 0.46 during the RCP8.5 run. The mean surface air temperature during Oct-Dec helps to control the heat flux from ocean to atmosphere as a result of the temperature difference across the ocean-atmosphere interface. Including this climate variable as a second regressor improves the fit in the 20C run to *R*^2^ = 0.78 and to *R*^2^ = 0.61 in the RCP8.5 run.

The growth rate of sea ice on the other hand is controlled—in the absence of incoming shortwave radiation—by the balance of heat fluxes at its base^51^. The growth of sea ice of thickness, *h*_*i*_ through time, *t* depends on a greater conductive heat loss to the atmosphere through the ice, *F*_*c*_ than heat flux from the ocean at its base, *F*_*w*_, as shown in Eq. 2, where *ρ*_*i*_ is the density of ice, *L*_*i*_ is the latent heat release/uptake on freezing/melting, and fluxes are positive upwards.

2 $${\rho }_{i}{L}_{i}\frac{d{h}_{i}}{dt}\,=\,{F}_{c}\,-\,{F}_{w}$$

As sea ice thickens, the conductive heat fluxes *F*_*c*_ through the ice diminish, bringing the growth rate down towards zero, depending on the size of the oceanic heat flux, *F*_*w*_. Other factors besides ice thickness also control *F*_*c*_, including the air temperature and snow cover. Assuming a layer of snow with thickness, *h*_*s*_ overlying ice with thickness, *h*_*i*_, with corresponding thermal conductivities *λ*_*s*_ and *λ*_*i*_, respectively, and linear temperature profiles through both layers, the conductive heat flux is

3 $${F}_{c}\,=\,\frac{\Delta T}{\frac{1}{k}\,+\,\frac{{h}_{i}}{{\lambda }_{i}}\,+\,\frac{{h}_{s}}{{\lambda }_{s}}}$$

where *k* is an effective heat transfer coefficient between the snow/ice surface and the atmosphere, and Δ*T* = *T*_*w*_ − SAT; in which *T*_*w*_ is the freezing temperature of water and SAT is the surface air temperature. Combining Eqs. 2, 3 and rearranging yields

4 $$\frac{d{h}_{i}}{dt}\,=\,\left(\frac{\Delta T}{\frac{1}{k}\,+\,\frac{{h}_{i}}{{\lambda }_{i}}\,+\,\frac{{h}_{s}}{{\lambda }_{s}}}\,-\,{F}_{w}\right)/{\rho }_{i}{L}_{i}$$

In the Arctic Ocean, the strong surface stratification suppresses upwards heat fluxes from the relatively warm Atlantic Water layer: *F*_*w*_ is generally low during the freezing season, of order 1 Wm^−2^ or less in the basin interiors, but sometimes higher directly over the Atlantic Water boundary current or over rough topography^52^. Unlike in the Southern Ocean, where *F*_*w*_ is on the order several tens of Wm^−2^, these low heat fluxes are generally no impediment to the growth of first year ice^51,53^.

### A linear model for ice production using internal variability

By revealing that the number of freezing area days and mean winter growth rate exhibit quite different characteristics, the decomposition in Fig. 4 offers a high-level insight into the competing processes governing ice production changes. We now seek to isolate a set of observable variables that can capture the physics of these processes. We then explain the changes in ice production through time with a linear model involving these variables.

As discussed above, variability in the number of freezing area days during Oct-Apr is dominantly captured by variation in the Sep sea surface temperature. The mean growth rate, meanwhile, must be controlled by the variables affecting the heat fluxes *F*_*w*_ and *F*_*c*_ (Eq. 2). We can diagnose *F*_*w*_ directly from CESM-LE and observations (though the latter are sparse), but we seek to capture *F*_*c*_ in terms of readily observable climate variables, using insight from Eq. 3.

The conductive heat flux is inversely dependent on snow and ice thicknesses (Eq. 3). Since snow is much more insulating than ice (*λ*_*s*_ ≈ 0.1*λ*_*i*_), even a thin layer of snow may significantly affect the conductive heat flux^51^. Equation 3 also shows that as surface air temperatures rise—and Δ*T* lessens—the conductive heat flux decreases in linear relation, as does the growth rate (Eq. 4). Identified positive correlations between surface air temperatures and growth rate^32^ rest on a causality in the opposite direction: increased growth rates and attendant upwards heat fluxes serve to boost surface air temperatures locally. We must therefore include the effect of air temperature in a way that explicitly captures its causal impact on sea ice growth. We do this by mimicking the growth rate equation: we include Δ*T* as a factor in each of our regressors.

The sea ice thickness *h*_*i*_ features on both sides of the growth rate equation (Eq. 4) due to its involvement in *F*_*c*_. To avoid circular logic we must not include the time evolution of *h*_*i*_ during winter in the regressors of the linear model. However, we can consider initial conditions from which *h*_*i*_ evolves. These initial conditions can be both the ice thickness at the start of the season, and occasions during the season when *h*_*i*_ is set to zero (by divergence) as an initial condition for growth. The minimum sea ice area (usually occurring in September) determines the area over which *h*_*i*_ = 0 at the start of the season: the Sep open water area. In addition, open water is created near-continually throughout the freezing season by divergence. Under tension, ice cracks in a brittle manner rather than thinning; the open water area is equal to the area diverged.

Divergence of the Arctic ice pack is spatially and temporally complex, and to understand its effect on thermodynamic growth we must also consider the role of convergence, which thickens ice through ridging and rafting, and may partially balance divergence that occurs in a given area. Because the growth rate of ice is an inverse function of its thickness (Eq. 3), and because ice cracks under tension—thus setting *h*_*i*_ = 0 in the diverged area—we can expect the effects of convergence and divergence on growth rate to be asymmetric. We must therefore separately consider not only net divergence over the region, but also divergence that is balanced by convergence elsewhere within the region: we term this the “compensated” divergence.

We thus classify three settings in which open water is made available for ice growth: the Sep open water, created by summer melting and ice advection; the open water area due to net divergence; and the open water area due to compensated divergence. The sea ice growth in open water areas will be linearly related to Δ*T* and to the area that has been opened. In our linear model, we neglect the timing of divergence and simply use the total area diverged during the winter season in both cases.

We can include the effect of snow as Δ*T*/*h*_*s*_, as per Eq. 3, where *h*_*s*_ is the mean winter snow depth wherever ice is present. Gathering together these terms, our linear model for total winter ice production, including regression coefficients, *β*_*n*_ is then:

5 $$\,{{\mbox{Ice Prod}}}\,=\,{\beta }_{1}\Delta T/{h}_{s}\,+\,{\beta }_{2}\Delta T{A}_{Sep}\,+\,{\beta }_{3}\Delta T{A}_{net}\,+\,{\beta }_{4}\Delta T{A}_{comp}\,+\,{\beta }_{5}{{{{{{{{\rm{SST}}}}}}}}}_{Sep}$$

Where *A*_*Sep*_ is the open water area available in September, *A*_*net*_ is the net area diverged integrated over the winter season, *A*_*comp*_ is the compensated area diverged integrated over the winter season, SST_*Sep*_ is the Sep sea surface temperature (the top 10 m ocean temperature), and Δ*T* is the difference between the freezing temperature of seawater and the surface air temperature. We neglect the ocean-to-ice heat flux term, *F*_*w*_, simply because when it is included as an additional term in this multiple linear regression, the resulting regression coefficient is statistically insignificant (standardised *β* = 0.0006 and *p* value = 0.26 over the full run): *F*_*w*_ has no significant impact on ice production in the Kara-Laptev region in CESM-LE.

**Fig. 5:**
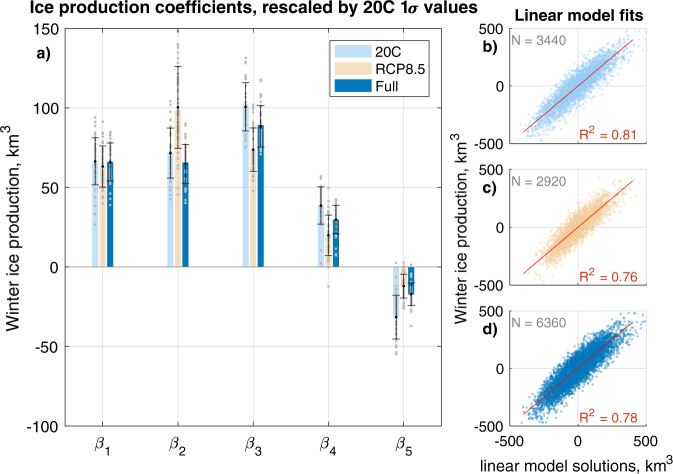
A linear model for ice production. **a** Winter ice production regression coefficients, standardised by the respective one standard deviation values in the 20C run. Coloured bars show the regression coefficients calculated using internal variability across all 40 ensemble members, with the ensemble mean removed, in the 20C (light blue), RCP8.5 (orange) and full (dark blue) datasets, respectively. Grey points indicate the regression coefficients as calculated using the deviations from the ensemble mean in individual models and indicate uncertainty. Black dots indicate the mean of the 40 estimates and whiskers indicate one standard deviation from the mean. Scatter plots on the right hand side illustrate the fit between the actual deviation of winter ice production values in all 40 models from the ensemble mean (y-axis) and the corresponding estimates from the linear model. **b** Regression model for 20C data alone, *R*^2^ = 0.81, associated with the regression coefficients shown in light blue in (**a**). **c** Regression model for RCP8.5 data alone, *R*^2^ = 0.76, associated with the regression coefficients shown in light orange in (**a**). **d** Regression model for the combined 20C and RCP8.5 datsets (‘full’), *R*^2^ = 0.78, associated with the regression coefficients shown in dark blue in (**a**).

We perform multiple linear regression across all ensemble members of CESM-LE to determine *β*_*n*_, using the departures of regressors and regressands in each ensemble member from ensemble-mean values, i.e. the internal variability, during the 20C run, RCP8.5 run and the full timeseries (Fig. 5). We plot standardised coefficients, by dividing by 20C run standard deviation values for each regressor. This indicates which regressors have the largest effect on ice production in terms of the natural range of variability in the 20C run. We preserve this standardisation in the plots for the RCP8.5 run and the full timeseries coefficients in order to compare absolute magnitudes. The variance in the CESM-LE internal variability of ice production explained by the linear model is consistently high: 81% during the 20C run, 76% during the RCP8.5 run and 78% over the full run (Fig. 5b, c, d). All *p* values for the different terms are <10^−30^ and the *p* values for the linear model solutions as a whole are <10^−300^.

We limit the dataset to individual ensemble members (grey points, Fig. 5) to estimate the uncertainty associated with these coefficients. The means of these clusters (black points) are very close to the values computed using the whole ensemble (bars). The one standard deviation windows of the individual ensemble estimates are indicated with black whiskers and provide an estimate of the uncertainty associated with each regression coefficient.

The coefficients from the 20C, RCP8.5 and full timeseries show overlapping uncertainty windows in all cases, and unanimous agreement in sign. The absolute magnitude of the *β*_1_ coefficient is remarkably similar between runs (Fig. 5), suggesting that the role of Δ*T*/*h*_*s*_ in determining ice production is relatively unchanging. While there are differences in magnitude in other coefficients, the relative consistency under the different climate conditions presented by 20C and RCP8.5 suggests that the linear model is physically robust.

Three regressors are dominant in their contribution to the internal variability: net divergence × Δ*T* (*β*_3_), Sep open water × Δ*T* (*β*_2_), and inverse snow depth × Δ*T* (*β*_1_). This reinforces the important role of variability in wind-driven sea ice divergence in explaining year-to-year changes in ice production in the Kara-Laptev region^36^.

### Explaining forced changes in ice production

**Fig. 6:**
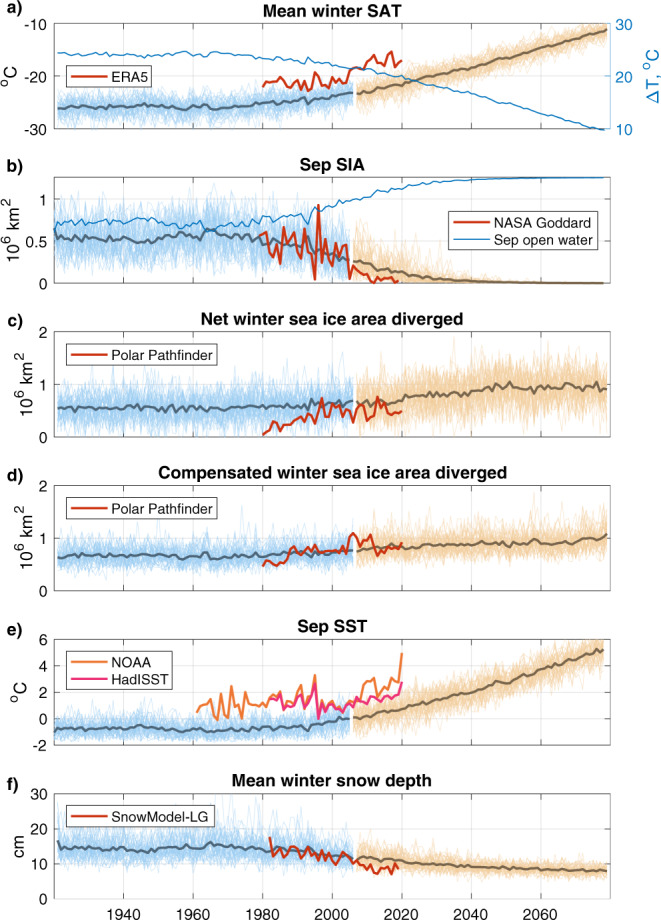
Key climate variables. Timeseries of climate variables from CESM-LE used in the linear model, with observation-based estimates superimposed in red (from ERA5, NASA Goddard, Polar Pathfinder, NOAA OI SSTv2, HADISST and SnowModel-LG). All 40 ensemble members shown in light blue (20C) and orange (RCP8.5). Ensemble mean indicated by darker lines. **a** Surface air temperatures, CESM-LE ensemble-mean Δ*T* in blue; (**b**) Sep sea ice area (SIA), CESM-LE ensemble-mean Sep open water area (*A*_*Sep*_) in blue; (**c**) net area diverged; (**d**) compensated area diverged; (**e**) September sea surface temperature (Sep SST); (**f**) Snow depth on ice.

Having seen that our linear model can explain a large fraction of the internal variability in winter sea ice production across all ensemble members of CESM-LE, we now ask whether it can explain the forced changes in sea ice production associated with climate change, and, in doing so, help us to understand the process at play. The linear model reconstruction of these forced changes is determined by the trends in the ensemble-mean values of the climate variables used (Fig. 6) and the corresponding regression coefficients, which we take from the internal variability (Fig. 5).

**Fig. 7:**
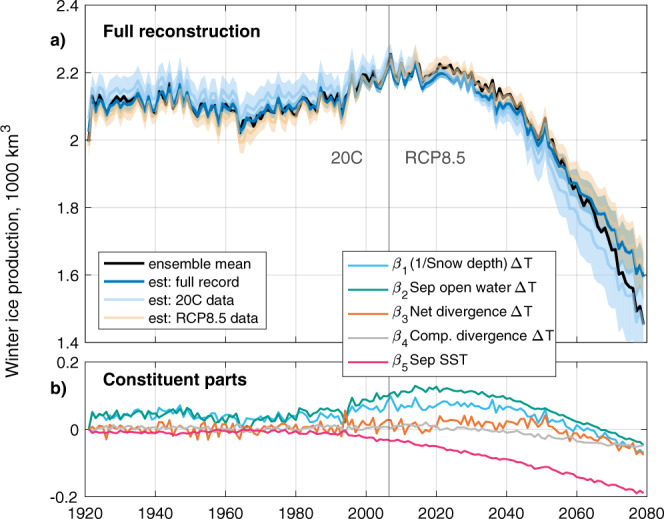
Application of linear model to forced changes in ice production. **a** Ensemble-mean winter sea ice production in CESM-LE (black line), and reconstruction of ensemble mean using regressions trained on internal variability over the 20C run (light blue line), RCP8.5 run (light orange line) and full 1920–2080 timeseries (heavy blue line). Uncertainty windows derived from 40 reconstruction estimates using the regression coefficients associated with each model run (grey dots, Fig. 5): the window is the 1 standard deviation envelope. **b** Contribution to the reconstruction made by each component of the linear model, when trained upon internal variability from the full timeseries, plotted relative to the first winter (1920/1921).

We construct timeseries of ice production using the regression coefficients calculated from internal variability in (a) 20C data only, (b) RCP8.5 data only and (c) the full timeseries (Fig. 7a). All three solutions for sea ice production successfully reconstruct the overall shape and interannual variability in the ensemble mean over the full period. The reconstructions also accurately capture the timing of the turning point from rising to falling ice production.

In light of the success of the linear model at reconstructing forced changes in sea ice production, we can next interrogate the contributions made by each term in the linear model (Fig. 7b). Interpretation of these trends should critically consider that each regressor, except for Sep SST, contains the product of surface air temperatures (as Δ*T*) and another climate variable. The trends of the underlying timeseries (Fig. 6) provide useful context.

The greatest contribution to increasing ice production up to 2020 comes from the *β*_2_Δ*TA*_*Sep*_ term. This term can be interpreted as ice produced in the open water available at the start of the freezing season. As the Sep open water area increases during 1970–2020 (Figs. 2a, 6b), this term contributes increasing ice production, despite SAT warming. As the ensemble mean Sep sea ice area approaches zero at around 2020 (Fig. 6b), this negative feedback on sea ice loss approaches a limit. From this point, the open water area, *A*_*Sep*_ is fixed to the total study area because the region is ice free. Meanwhile, Δ*T* continues to fall (as SAT rises), resulting in a negative trend in the ice production attributable to the Sep open water setting.

Decreasing snow thickness on sea ice also provides an important contribution to rising ice production between approximately 1990 and 2010 (Fig. 7b). While snow continues to thin to 2080 (Fig. 6f), after c. 2030 changes in the *β*_1_Δ*T*/*h*_*s*_ term are dominated by warming SAT, leading to a decline in ice production from this term in spite of the thinning snow cover.

While the Δ*TA*_*net*_ regressor has the largest standardised coefficient (*β*_3_, Fig. 5a) and is therefore the leading contributor to internal variability, the balance between the forced trends in Δ*T* and *A*_*net*_ in the CESM-LE ensemble-mean result in a modest contribution to the forced changes in ice production from this term. Similarly, *β*_4_Δ*TA*_*comp*_ shows little contribution to forced changes. In both cases, this balance between warming and increasing divergence holds out until the mid-21st Century, before warming air temperatures begin to dominate, leading to a decline in ice production attributable to these terms.

The *β*_5_SST_*Sep*_ term, though showing the weakest standardised regression coefficient in the internal variability space, is the dominant driver of the forced decline in ice production. Indeed, Sep SST exhibits a clear warming trend in the RCP8.5 run that rapidly departs from the range of 20C internal variability (Fig. 6e). There is evidently, then, a strong role for ocean heat derived from summer heat uptake in delaying the freezing season and thus decreasing ice production. But is there a role for ocean heat in changing growth rate or ice production once the freezing season has begun? Ocean-to-ice heat fluxes (*F*_*w*_) in the region are generally low in CESM-LE: the ensemble-mean values are c. 0.5 W/m^2^ during 20C, rising throughout RCP8.5 to nearly 2 W/m^2^ by 2080. This, however, does not appear to noticeably impede ice growth in CESM-LE: *F*_*w*_ is statistically insignificant when included as an additional regressor in the linear model for ice production.

### Application to the observed Arctic

**Fig. 8:**
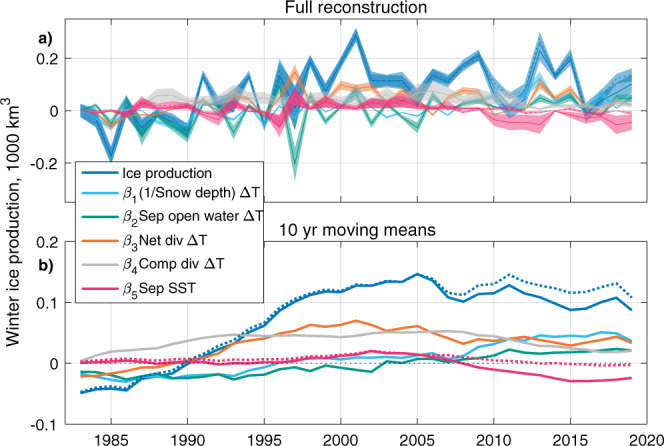
Application of linear model to historical changes. Changes in winter ice production in the Kara-Laptev seas plotted relative to the first winter of the reconstruction (1982/1983). The estimate uses climate variables from observation-based estimates and the linear model trained on CESM-LE internal variability. Data are plotted with respect to the new year of the winter in question. Dashed line is the result of using NOAA OI V2 SST product, while the solid line uses HadISST. Shaded uncertainty windows are derived from the one standard deviation spreads of the corresponding estimates using the 40 sets of regression coefficients from individual models (see Fig. 5). Full interannual evolution is plotted in (**a**) and 10-year moving means are plotted in (**b**). ‘Div’ is short for divergence; ʻcomp’ for compensated.

We next estimate changes in ice production in the Kara-Laptev region over recent decades (Fig. 8) by applying our linear model to observation-based estimates of the relevant climate variables (which can be seen in Fig. 6). We use regression coefficients derived from internal variability over all ensemble members of CESM-LE (details in Methods). The approach is therefore based on the assumption that ice production in the Kara-Laptev region has similar sensitivity to the five regressors in our linear model as it does in CESM-LE.

The resulting estimate of ice production based on observation-based climate data can be assumed to contain both internal and forced variability (Fig. 8a). Internal variability may be present on a range of timescales, but is most obvious on the interannual timescale, on which we may identify causes for the variation through examination of the regressors and individual climate variables; e.g. the recent warm winters of 2015/2016 and 2016/2017^13,29,30^ trigger relatively low ice production. The 10-year running mean (Fig. 8b), however, exhibits similar features to the ensemble-mean trend in CESM-LE: reconstructed winter ice production increases by 150–200 km^3^ from 1983 to 2000. From about the mid-2000s, ice production appears to show a slight decline. While uncertainty is difficult to constrain from the underlying observation-based estimates, we note that the reconstruction is somewhat sensitive to the choice of product for SST, especially from 2006/2007 onwards.

Unlike in CESM-LE, the *β*_2_Δ*TA*_*Sep*_ term is not the dominant driver of the increase in estimated ice production. We can reconcile this by noticing the anticorrelation (*R* = −0.77) between the Sep SIA and SAT timeseries (Fig. 6a, b). Both timeseries show marked jumps in the winter 2005/2006—these changes act in opposing directions in terms of ice production and thus tend to cancel one another out. Given that the Sep open water area has already reached a maximum (as Sep SIA has declined to zero in recent years), we can expect future atmospheric warming to yield a negative ice production trend from this term, as per Fig. 7b.

Examining the trends from the other terms, we see that Sep SSTs exert a negative influence on ice production from c. 2010. The *β*_1_Δ*T*/*h*_*s*_ term causes marked interannual variability and a strong positive trend in ice production: snow thinning outcompetes warming over this period.

The contributions to ice production from the two terms involving divergence exhibit the strongest increases relative to the first year of the timeseries, out-competing atmospheric warming, before plateauing or shallowly declining in the final c. 15 years. These terms play a larger role than in the forced changes seen in CESM-LE (Fig. 7b). This is related to the steeper trends in ‘net’ and ‘compensated’ divergence from Polar Pathfinder over the observational window than the CESM-LE ensemble mean (Fig. 6c, d; SAT also plays a part in these terms).

## Discussion

CESM1.1 is a highly complex coupled climate model; however, we have shown that we can understand internal variability and forced trends in ice production in the Arctic Ocean’s “ice factories" using a simple linear model. The linear model, which uses insight from the growth rate equation, captures a very high degree of variance in the internal variability of ice production across CESM-LE, is robust to a range of climate conditions, and successfully reconstructs the forced rise-then-fall behaviour of ice production in the Kara-Laptev region in this model. Previous studies have identified rise-then-fall behaviour and corresponding changes in the statistical relationships between ice production and climate variables. Our study builds on this by explaining both the rise and the fall in ice production in terms of consistent underlying physical processes. Our analysis shows that a number of negative feedbacks to ice loss—increasing Sep open water area, increasing divergence, reducing snow depth—contribute to a gradual rise in ice production in CESM-LE from c. 1970–2010, but are increasingly outcompeted by atmospheric warming through the 21st Century under the RCP8.5 emissions pathway (Fig. 8b). Together with an important contribution from increasing upper ocean temperatures at the end of summer, this leads to a decline in ice production. A greater reservoir of oceanic heat at the end of summer requires more time to cool, thus shortening the freezing season (reducing the “usage” of the ice factory). Sep upper ocean temperatures can be expected to continue to rise as a result of increasing absorption of solar radiation (due to reducing summer sea ice concentration), increasing radiative forcing, and increasing (and warming) river runoff ^54,55^.

In CESM-LE, ice production in the Kara-Laptev region passes a peak around 2020. Our analysis shows that the timing of peak ice production is primarily set by the timing at which Sep sea ice area in the Kara-Laptev region approaches zero. At this point, one of the leading negative feedbacks on the loss of ice—the expansion of the Sep open water area—has reached a limit and can no longer contribute additional ice production in this region. Continued atmospheric warming then ensures a decrease in the ice production attributed to this setting. In observational data, the expansion of the Sep open water area has also reached a limit in recent years. As such, we have good reason to expect ice production in the Kara and Laptev seas to decline in the coming decades under continued greenhouse forcing. As it is, our estimate of historical ice production suggests that ice production is currently passing a peak in the Kara-Laptev seas, and we can expect a marked fall in ice production to follow under further climate warming.

Declining ice production in the Kara-Laptev seas raises a number of wider implications. Sea ice from the Kara and Laptev seas carries sediment, pollutants, trace elements and gases into the central Arctic and beyond^36,56,57^. Falling sea ice production in the region will therefore affect redistribution of biogeochemical matter in the Arctic Ocean, with implications for primary production and biodiversity^56^. Shifting patterns of ice production also raise important questions for the stability of the Arctic halocline, which limits upwards heat fluxes from Atlantic Waters and restricts the depth of water that is convectively cooled prior to and during sea ice growth^58^. High rates of freezing in the Kara and Laptev seas strengthen the cold halocline of the central Arctic basins via advective interleaving of cold shelf waters, densified by brine rejection, into the halocline of the Eurasian Basin^44,59^. Another mechanism affecting the halocline involves the injection of cold and fresh waters into surface waters of the interior basins in summer from the melting of shelf-derived sea ice, which renew the cold halocline via a ‘convective mode’ during winter convection^45,60^. Both mechanisms may operate in different seasonal and spatial contexts^38,61,62^, and both depend on ice production on the shelves. Meanwhile, increasing ice production and brine rejection in the Eurasian basin interior may weaken the halocline by encouraging vigorous convection, allowing heat from the Atlantic Waters to reach the surface^47,49^. Our linear model provides good reasons to expect that ice production in the interior basins may yet rise further before falling: as the Sep sea ice edge progressively retreats through the central Arctic (Fig. 2a), we can expect year-on-year increases in Sep open water area and sea ice divergence, and decreases in snow depth in these regions—all of which drive increasing ice production. Indeed, in CESM-LE, the share of Kara-Laptev ice production in the Arctic Ocean (excluding Barents Sea) declines from 25% in the 20C run to 19% by 2080 (it comprises 16% of the area). Reduced ice production in the Kara and Laptev seas will alter the physical and biogeochemical makeup of the Arctic Ocean, and we suggest that future work should address the profound impact of these foreseeable changes.

A strength of the linear model is that it is simple, yet still captures the vast majority of variance in internal variability in CESM-LE, and is robust over a range of climate conditions. This simplicity makes it easy to extend the linear model to other sources of data, such as from observations or other climate models, and provides clear physical insight. Adding complexity, by e.g. considering the timing of divergence events, or analysing spatial variations, could add further skill, and further increase the robustness to different climate conditions. However, it would come at the expense of simplicity.

Our methodology could be applied to other regions in the polar oceans and other climate models. While much of the process-based understanding we have developed for the Kara-Laptev region is transferable to other areas, there are important differences in the mean sea ice state and oceanic setting that affect the balance of *F*_*c*_ and *F*_*w*_. As ice is generally thin in the Kara and Laptev seas, owing to the wind-driven time-mean divergence of sea ice, and because little ice survives the summer, *F*_*c*_ is comparatively large (Eq. 3). On the other hand, once the excess heat stored in the upper ocean has been removed to the atmosphere, *F*_*w*_ is low. In the Kara and Laptev polynyas, winter convection may propagate to the seafloor^33,40^, but because Atlantic Waters rarely upwell into these shallow areas where freezing is most intense, future increases in *F*_*w*_ are likely to be limited, though Atlantic Waters may warm halocline waters that intrude onto the shelf ^63^. While in our linear model for CESM-LE *F*_*w*_ is an insignificant term in the Kara-Laptev region, it may be significant in other areas of the polar oceans (and perhaps in other models in the same region), and could be included as an additional regressor. Sea-ice thicknesses at the start of winter (as an initial condition) can additionally be included as an additional regressor (as Δ*T*/*h*_*i*_). We did not include this term because observational estimates of sea ice thickness are much more time-limited than the other variables we use, precluding a multi-decadal observation-based estimate of ice production. Further, its inclusion only marginally increased the skill of the linear model. However in other regions this term may be more important. Application of this technique to other regions and models will help to build physical understanding of the controls on sea ice production, and motivate understanding of the impact of future changes in ice production.

## Methods

### Regional and methodological choices

There is observational evidence for an Arctic-wide increase in ice production. Here, however, we focus on one region which shows this rise-then-fall behaviour in CESM-LE. Note, however, that other climate models may not necessarily show a rise-then-fall in ice production in this same region. We adopt a regional perspective because the growth conditions of sea ice across the Arctic are highly regionally variable, and focus on one region with fairly consistent characteristics: the Kara and Laptev seas. Moreover, these shelf seas have special status due to their outsized contribution to Arctic sea ice production.

Analysis of ice production from satellite data is possible with a combination of sea ice thickness, concentration and velocity data. However, we do not perform it here for two main reasons: a) the satellite record of sea ice thickness is very short, precluding the development of robust statistical relationships; b) there is a large amount of uncertainty in sea ice thickness measurements where ice is thin^64,65^, precluding the regional investigation we undertake here, though not undermining the Arctic-wide evidence for increasing sea ice production described in the Introduction.

### Climate model

To analyse the forced response of sea ice production in the Kara and Laptev seas to climate change, we use data from all 40 ensemble members of the Community Earth System Model Large Ensemble (CESM-LE)^66^. A large ensemble such as CESM-LE permits investigation of forced responses to climate change in the context of internal climate variability^66^. We require an ensemble approach in our methodology in order to develop a linear model based on internal variability that we then use to reconstruct forced changes. CESM-LE is based on the fully coupled model CESM1.1, and comprises the Community Atmosphere Model, version 5 (CAM5); the Parallel Ocean Programme, version 2 (POP2); the Community Land Model, version 4; and the Los Alamos Sea-Ice Model CICE, version 4 as its sea ice component. The version of CICE used features improvements to shortwave radiation interactions, including the effects of melt ponds and aerosol deposition on ice^67^. The spatial resolution of the CESM1.1 ocean and sea ice models is nominally 1° × 1° longitude by latitude, while the atmospheric model is 0.9° × 1.25°.

All CESM-LE ensemble members are forced by the same external forcing data, separated into two runs. Firstly, a run from 1920 to 2005 forced by historical external forcing data^68^ that we refer to as the 20C run; secondly, from 2006 to 2100, a high-emissions RCP8.5 run^69^, leading to over 4 ^∘^C warming by 2100. The ensemble spread is entirely generated by simulated internal climate variability originating from very small, random differences in the initial air temperature fields. Here we rely on monthly data between 1920–2080, sufficient time to assess major forced changes in ice production in the Kara and Laptev seas.

Although recent emissions have tracked the RCP8.5 emissions scenario^70,71^, the Intergovernmental Panel on Climate Change (IPCC) consider it a scenario of low likelihood in their sixth assessment report (AR6), due to recent developments in the energy sector^72^; in the context of currently pledged climate and energy policies more plausible scenarios project between 2 °C and 3 °C by 2100^73^. Despite this, the RCP8.5 emissions scenario retains great value for fundamental science: it provides a large warming signal, which is useful for interpreting climate dynamics and relationships in the climate system in the presence of noise.

Coupled climate models of the CMIP5 and CMIP6 generations exhibit substantial biases in aspects of their representations of the Arctic Ocean, particularly in the temperature of the Atlantic Water layer, and the salinity of the halocline and surface fresh layer^74,75^. While CESM1.1 and CESM-LE have their own biases, they have been used in a broad swathe of Arctic climate studies and generally show a serviceable correspondence to (sparse) observations^32,76–83^. Arctic sea ice thickness in CESM-LE broadly corresponds in spatial mean pattern and trend during 1980–2015 with PIOMAS^84^. On an Arctic-wide basis it exhibits thicker (order several 10 cm) sea ice than PIOMAS, though the differences versus PIOMAS are smaller and vary either side of zero spatially in the Kara-Laptev region^32,85^. Although the satellite record of sea ice thickness is short, CESM-LE compares well with estimates of inter-seasonal thickness changes and interannual variability^32^. Declining Arctic sea ice concentration and extent in CESM-LE has been more comprehensively compared to observations, which fit within its ensemble spread and compare well across all seasons^77,80,81^. The changing open water season also shows correspondence with satellite observations, including in the Laptev Sea^81^.

### Climate variables computed

To capture winter thermodynamic sea ice production, we use monthly data from October to April, inclusive, as per Petty et al.^32^. In contrast with Petty et al. we analyse freshwater exchanges with the ocean, rather than changes in ice thickness, to explicitly isolate thermodynamic changes. Sea ice production is calculated as the sum of only the ocean-to-ice part of thermodynamic ice-ocean freshwater fluxes (it does not include melting).

Our calculation of freezing area days using CESM-LE data is an approximation, dependent on the spatial discretisation of the ocean and sea ice components (nominally 1° × 1°), temporal averaging (monthly) and number of ensemble members used (40). The mean winter growth rate in all such freezing grid cells is given by the total winter ice production divided by the number of freezing area days.

Climate variables are either explicitly taken in September (SST and open water area), or calculated as a mean (snow depth, surface air temperature) or sum (divergence) throughout the winter period of October to April. All variables are computed within the study region shown in Fig. 1 and are appropriately area-weighted using the relevant cell areas. The Kara-Laptev region is defined by the coastline and the following (lat,lon) vertices: (72.5, 70), (82, 70), (82, 110), (78, 110), (78, 150), (72, 150).

The variables that contribute to the regressors are as follows. (1) Winter mean snow depth on sea ice, averaged over all cells with more than <15% sea ice. (2) Sep sea ice concentration, converted to sea ice area using grid cell areas. (3) Sea-ice area diverged throughout the winter, (a) summed over all grid cells where ice is present (net divergence), (b) summed over cells in which cells are divergent only (positive divergence). (4) Winter mean surface air temperatures over the whole oceanic region. (5) Sep ocean temperatures averaged across the whole oceanic region in the top (10 m thick) grid cell.

We investigate two types of sea ice divergence. Firstly, the net divergence, which is area-integrated divergence, including convergence. We are also interested in divergence within the region that is compensated by convergence somewhere else in the region; this divergence can open or expand leads but still not lead to net divergence. To capture this compensated divergence, we record the “positive divergence”, which is counted only over cells where the divergence is positive. The compensated divergence is then found as compensated div = positive div − net div. There is therefore no double counting between net and compensated divergence. The two are therefore only weakly correlated—preferable for the purposes of building a linear model. The divergence measures are converted to areas diverged before inclusion in the linear model for ice production.

Surface air temperature is converted to Δ*T* by taking the difference between the freezing temperature of seawater, −1.8 °C, and the surface air temperature. Thus, the colder the surface air temperature, the larger and more positive Δ*T*. Sep open water area is derived by taking the difference between the total oceanic area of the region and the Sep sea ice area.

Data are plotted according to the second year in each winter, e.g. winter 2015/2016 is labelled 2016. The variables extracted from the observation-derived estimates are computed in a consistent manner to their equivalents from CESM-LE.

### Deriving the linear model regression coefficients

We derive the regression coefficients for the linear model by using the internal variability in all 40 ensemble members of CESM-LE. The regressand is total winter ice production in the Kara and Laptev seas, and the regressors are: (1) Δ*T*/*h*_*s*_ (2) Δ*TA*_*Sep*_ (3) Δ*TA*_*net*_ (4) Δ*TA*_*comp*_ (5) SST_*Sep*_. In each case these are deviations from the ensemble mean for each year, such that the linear model is centred around zero. Through multiple linear regression, we derive the associated regression coefficients, *β*_*n*_ that minimise the least squares difference of the linear model to the original ice production values. In Eq. 6, the superscript *e* indicates that the variable is taken from all ensemble members, and for all years under consideration.

6 $${{{\mbox{Ice Prod}}}}^{e}\,=\,{\beta }_{1}\Delta {T}^{e}/{h}_{s}^{e}\,+\,{\beta }_{2}\Delta {T}^{e}{A}_{Sep}^{e}\,+\,{\beta }_{3}\Delta {T}^{e}{A}_{net}^{e}\,+\,{\beta }_{4}\Delta {T}^{e}{A}_{comp}^{e}\,+\,{\beta }_{5}{{{{{{{{\rm{SST}}}}}}}}}_{Sep}^{e}$$

The standardised values of the coefficients are shown in Fig. 5. We construct the model three times, based on data from the 20C run, the RCP8.5 run, and the combined 20C and RCP8.5 runs (‘full’).

### Uncertainty estimates

While we can find the root mean square error associated with the linear model, we view a more useful estimate of uncertainty as that derived from the spread of estimates when limiting the dataset to single ensemble members. This connects the methodology to the constraints of a single climate realisation, which is all we may observe, and all we have from many climate models. To do this, we subtract the ensemble mean from each model realisation for each climate variable (though we note that a decadal moving mean could alternatively be used when there is no ensemble mean to compare against). We then compute the regression coefficients as before, but with a dataset that is 1/40th of the size of the whole ensemble estimates. All 40 resulting versions of each regression coefficient are displayed as grey dots in Fig. 5a. We take the mean of these 40 estimates and plot it as a black dot, and plot the one standard deviation window using black whiskers. The mean estimates of the ensemble of estimates are very close to the estimate from the whole ensemble. Note that the root mean square errors associated with the linear regression using the whole ensemble are significantly smaller than the one standard deviation windows derived from the spread of individual ensemble member estimates.

### Reconstructing ice production

To reconstruct the ensemble-mean ice production in CESM-LE, we use regression coefficients derived from internal variability across the whole ensemble, but use climate variables from the ensemble mean. This is illustrated in the equation below: *β* coefficients are derived from internal variability across the whole ensemble as per Eq. 6, whereas superscript *m* denotes that the variable is from the ensemble mean. Superscript *m *recon indicates that the ice production timeseries is a reconstruction of the ensemble mean.

7 $${{{\mbox{Ice Prod}}}}^{m\,{{{{{{{\rm{recon}}}}}}}}}\,=\,{\beta }_{1}\Delta {T}^{m}/{h}_{s}^{m}\,+\,{\beta }_{2}\Delta {T}^{m}{A}_{Sep}^{m}\,+\,{\beta }_{3}\Delta {T}^{m}{A}_{net}^{m}\, \\+\,{\beta }_{4}\Delta {T}^{m}{A}_{comp}^{m}\,+\,{\beta }_{5}{{{{{{{{\rm{SST}}}}}}}}}_{Sep}^{m}\,+\,{{{{{{{\rm{const}}}}}}}}$$

As our linear model is trained on internal variability, which numerically is comprised of deviations either side of zero because we subtract the ensemble mean, we add a constant to compare the reconstruction with the original ice production data. We choose the constant such that the mean of the reconstruction and the mean of the original ice production timeseries are the same.

### Observation-based estimates

We process the climate variables from observation-based sources for application with the linear model in the same manner as climate variables from CESM-LE.

We use monthly-mean 2 m temperatures from the ERA5 global reanalysis^86^, gridded at 0.25 ^∘^ × 0.25 ^∘^ resolution. ERA5 is the fifth generation of atmospheric reanalyses from the European Centre for Medium-Range Weather Forecasts (ECMWF). We use the terms surface air temperature and 2 m temperature interchangeably here. Surface air temperatures from ERA5 generally perform well in the Arctic relative to in situ observations, however consistently show warm biases of order several degrees over sea ice in winter months^87^. Our analysis of these data spans the winters 1979/1980 to 2019/2020.

To plot mean sea ice velocities and calculate sea ice divergence, we make use of the National Snow and Ice Data Centre (NSIDC) Polar Pathfinder v4.1 product, gridded at 25 × 25 km resolution and in weekly means^88^. Polar Pathfinder data are masked during processing to only provide vectors where ice concentration is >15%. We analyse data from Polar Pathfinder encompassing the winters 1979/1980 to 2019/2020.

To document changes in sea ice area, we use sea ice concentration data from passive microwave satellite retrievals, prepared by the NASA Goddard Space Flight Centre (NASA GSFC). We use the ‘merged’ data, which combine data derived from the Bootstrap and Nasa Team algorithms^89^, and compare well with other estimates^90,91^. We use data spanning the winters 1978/1979 to 2018/2019.

For snow depth, we use the output of a Lagrangian snow evolution model, SnowModel-LG, which provides daily data on a 25 km × 25 km grid. SnowModel-LG is forced by the MERRA-2 atmospheric reanalysis^92^ and the Polar Pathfinder ice motion vectors described above. The snow depth data are observations-based in the sense they are produced by observationally-informed atmospheric reanalysis and ice motion products. However we note that the snow depth data themselves should not be treated as observations. SnowModel-LG output compares well to observational data sets in spatial and seasonal variability of snow depth and density^93^. The data span winters 1981/1982 to 2018/2019. Consistent with our snow depth acquisitions from CESM-LE, we present snow thickness on sea ice where present.

For sea surface temperatures, we use the Met Office Hadley Centre’s sea ice and sea surface temperature (SST) data set, HadISST^94^. The monthly data are provided on a 1° × 1° grid. We use data spanning the winters 1961/1962 to 2019/2020. The SST data are taken from the Met Office Marine Data Bank (MDB), as well as data from the Comprehensive Ocean-Atmosphere Data Set (COADS) (now ICOADS) where there were no MDB data. The sea ice data are taken from a variety of sources including passive microwave retrievals and digitised sea ice charts. Additionally, we use monthly-mean SST data at a 1° × 1° resolution from the National Oceanic and Atmospheric Administration Optimum Interpolation Sea Surface Temperature v2 (NOAA OISSTv2) dataset^95^. We use NOAA OISSTv2 data from the winters 1981/1982 to 2019/2020.

To estimate historical ice production (superscript *o *estimate), we utilise our linear model and the observation-based climate variables (superscript *o*), as per

8 $${{{\mbox{Ice Prod}}}}^{o\,{{{{{{{\rm{estimate}}}}}}}}}\,=\,{\beta }_{1}\Delta {T}^{o}/{h}_{s}^{o}\,+\,{\beta }_{2}\Delta {T}^{o}{A}_{Sep}^{o}\,+\,{\beta }_{3}\Delta {T}^{o}{A}_{net}^{o}\, \\+\,{\beta }_{4}\Delta {T}^{o}{A}_{comp}^{o}\,+\,{\beta }_{5}{{{{{{{{\rm{SST}}}}}}}}}_{Sep}^{o}\,+\,{{{{{{{\rm{const}}}}}}}}$$

In order to just have one central estimate and one set of uncertainty windows, we pool all 40 estimates of the regression coefficients across the 20C, RCP8.5 and ‘full’ runs, and evaluate the linear model with each, yielding 120 estimates. We take the mean as the central estimate, and display the one standard deviation envelope associated with each term and the total ice production estimate (Fig. 8). The uncertainty associated with the 10-yr running mean (Fig. 8b) increases when the number of years averaged over is limited by the end of the time series. As Fig. 8b is a running mean of Fig. 8a, the timeseries do not begin on 0.

## Data Availability

The CESM-LE data^66^ used in this study are accessible at the NCAR/UCAR CESM website: https://www.cesm.ucar.edu/projects/community-projects/LENS/data-sets.html. ERA5 data^86^ are accessible via the Copernicus Climate Change Service portal (https://cds.climate.copernicus.eu/). Polar Pathfinder sea ice motion data^88^ are accessible at the NSIDC website (10.5067/INAWUWO7QH7B). The NASA GSFC sea ice concentration data^89^ are accessible at the NSIDC website (10.7265/N59P2ZTG). SnowModel-LG data^92,93^ are available at the NSIDC website (Liston, G. E., J. Stroeve, and P. Itkin; Lagrangian Snow Distributions for Sea-Ice Applications; 10.5067/27A0P5M6LZBI). HadISST data^94^ were obtained from https://www.metoffice.gov.uk/hadobs/hadisst/and are ⓒ Crown Copyright, Met Office, 2003, provided under a Non-Commercial Government Licence http://www.nationalarchives.gov.uk/doc/non-commercial-government-licence/version/2/. The NOAA OISSTv2 dataset^95^ is provided by the NOAA/OAR/ESRL PSL, Boulder, Colorado, USA, and accessible at their website (https://psl.noaa.gov/data/gridded/data.noaa.oisst.v2.html).

## Code availability

Codes used in this paper are available at https://github.com/samcornish/IceFactories.
